# Supplementary material for: Strong mnemonic prediction errors increase cognitive control, attention, and arousal
Source: eLife. 2026 Oct 21;15:RP109678. doi: 10.7554/eLife.109678 (PMC13686618; doi:10.7554/eLife.109678)
Supplement: Supplementary file 2 [file elife-109678-supp1.docx]

**SUPPLEMENTAL MATERIALS**

**Strong mnemonic prediction errors increase cognitive control, attention, and arousal**

Alice M. Xue^1,2^, Jenna Jokhani^1,2^, Anthony M. Norcia^1,2^, and Anthony D. Wagner^1,2^

^1^Stanford Department of Psychology

^2^Stanford Wu Tsai Neurosciences Institute

**Table A: Frontal theta trial-level regression model including miss and false alarm trials**

|  | **Start** | **End** | **Mean β [95% CI]** |
| --- | --- | --- | --- |
| Mismatch v. Match | 2.064 | 2.640 | 0.34 [0.23, 0.45] |
| Mismatch v. Match | 3.296 | 3.928 | -0.10 [-0.15, -0.05] |
| Incorrect v. Correct | 2.536 | 2.888 | 0.55 [0.16, 0.93] |
| Memory strength (Weak v. Strong) | 0.360 | 0.528 | -0.06 [-0.12, -0.01] |
| Memory strength (Weak v. Strong) | 2.520 | 2.880 | 0.17 [0.08, 0.27] |
| Mismatch effect x Memory strength | 0.776 | 0.848 | -0.15 [-0.30, -0.01] |
| Mismatch effect x Memory strength | 2.136 | 2.672 | -0.35 [-0.51, -0.18] |
| Mismatch effect x Memory strength | 3.032 | 3.096 | 0.12 [0.01, 0.24] |
| Mismatch effect x Memory strength | 3.384 | 3.488 | 0.11 [0.01, 0.20] |
| Mismatch effect x Memory strength | 3.640 | 3.816 | 0.12 [0.02, 0.23] |
| Mismatch effect x Accuracy | 0.536 | 0.776 | -0.26 [-0.47, -0.06] |
| Mismatch effect x Accuracy | 2.232 | 2.312 | -0.50 [-0.96, -0.05] |
| Accuracy x Memory strength | 2.640 | 2.808 | -0.41 [-0.76, -0.06] |
| Mismatch x Accuracy x Strength | 0.424 | 0.824 | 0.35 [0.15, 0.55] |
| Mismatch x Accuracy x Strength | 3.648 | 3.792 | -0.37 [-0.68, -0.05] |

**Table B: Posterior alpha trial-level regression model including miss and false alarm trials**

|  | **Start** | **End** | **Mean β [95% CI]** |
| --- | --- | --- | --- |
| Mismatch v. Match | 0.760 | 1.000 | -0.08 [-0.14, -0.02] |
| Mismatch v. Match | 2.040 | 2.104 | 0.07 [0.01, 0.13] |
| Mismatch v. Match | 2.576 | 3.064 | -0.14 [-0.20, -0.07] |
| Mismatch v. Match | 3.448 | 3.648 | -0.12 [-0.21, -0.02] |
| Incorrect v. Correct | 1.096 | 1.376 | -0.22 [-0.37, -0.08] |
| Incorrect v. Correct | 1.504 | 1.736 | -0.21 [-0.34, -0.07] |
| Incorrect v. Correct | 2.240 | 2.904 | -0.18 [-0.26, -0.09] |
| Incorrect v. Correct | 3.072 | 4.000 | -0.31 [-0.43, -0.19] |
| Memory strength (Weak v. Strong) | 0.376 | 0.520 | -0.06 [-0.10, -0.02] |
| Memory strength (Weak v. Strong) | 1.312 | 2.096 | 0.13 [0.06, 0.20] |
| Memory strength (Weak v. Strong) | 2.640 | 3.352 | -0.16 [-0.22, -0.09] |
| Mismatch effect x Memory strength | 0.840 | 1.016 | 0.11 [0.02, 0.20] |
| Mismatch effect x Memory strength | 2.648 | 3.128 | 0.16 [0.09, 0.24] |
| Mismatch effect x Accuracy | 1.088 | 1.280 | 0.30 [0.06, 0.54] |
| Mismatch effect x Accuracy | 2.736 | 2.904 | 0.29 [0.11, 0.46] |
| Accuracy x Memory strength | 1.072 | 1.272 | 0.28 [0.12, 0.43] |
| Accuracy x Memory strength | 2.488 | 2.896 | 0.18 [0.06, 0.29] |
| Accuracy x Memory strength | 3.096 | 3.224 | 0.22 [0.05, 0.39] |
| Accuracy x Memory strength | 3.440 | 3.640 | 0.25 [0.08, 0.43] |
| Accuracy x Memory strength | 3.752 | 4.000 | 0.34 [0.14, 0.54] |
| Mismatch x Accuracy x Strength | 1.032 | 1.240 | -0.42 [-0.70, -0.15] |
| Mismatch x Accuracy x Strength | 2.768 | 2.912 | -0.32 [-0.56, -0.09] |

**Table C: Pupil trial-level regression model including miss and false alarm trials**

|  | **Start** | **End** | **Mean β [95% CI]** |
| --- | --- | --- | --- |
| Mismatch v. Match | 2.27 | 2.87 | 0.15 [0.05, 0.25] |
| Mismatch v. Match | 3.29 | 3.94 | 0.18 [0.06, 0.31] |
| Incorrect v. Correct | 2.99 | 4.00 | 0.74 [0.37, 1.11] |
| Memory strength (Weak v. Strong) | 0.93 | 1.78 | -0.10 [-0.18, -0.03] |
| Memory strength (Weak v. Strong) | 2.61 | 2.82 | -0.12 [-0.22, -0.02] |
| Memory strength (Weak v. Strong) | 3.33 | 4.00 | 0.20 [0.08, 0.33] |
| Mismatch effect x Memory strength | 2.16 | 4.00 | -0.29 [-0.41, -0.17] |
| Mismatch effect x Accuracy | 1.61 | 4.00 | -0.55 [-0.91, -0.19] |
| Accuracy x Memory strength | 2.98 | 4.00 | -0.70 [-1.07, -0.33] |
| Mismatch x Accuracy x Strength | 2.07 | 2.49 | 0.54 [0.08, 1.00] |
| Mismatch x Accuracy x Strength | 2.68 | 4.00 | 0.72 [0.31, 1.13] |

**Table D: PC1 scores as a function of Mismatch and Strength**

| **Fixed effects** | **Estimate** | **CI** | **p-value** | **SE** | **DF** |
| --- | --- | --- | --- | --- | --- |
| (Intercept) | 0.038 | [-0.036, 0.113] | 0.312 | 0.038 | 112.302 |
| Mismatch | 0.032 | [-0.021, 0.085] | 0.242 | 0.027 | 10269.199 |
| Strength | -0.102 | [-0.147, -0.057] | 0.000 | 0.023 | 10271.457 |
| Mismatch x Strength | 0.027 | [-0.052, 0.106] | 0.506 | 0.040 | 10264.248 |

**Table E: PC1 scores and RTs**

| **Fixed effects** | **Estimate** | **CI** | **p-value** | **SE** | **DF** |
| --- | --- | --- | --- | --- | --- |
| (Intercept) | -0.655 | [-0.859, -0.452] | 0.012 | 0.025 | 1.234 |
| PC1 score | -0.050 | [-0.059, -0.040] | 0.000 | 0.005 | 170.709 |
| Strength | 0.201 | [0.185, 0.216] | 0.000 | 0.008 | 104.316 |
| Mismatch | 0.159 | [0.142, 0.176] | 0.000 | 0.009 | 136.383 |
| PC1 score x Strength | -0.004 | [-0.014, 0.007] | 0.479 | 0.005 | 9151.269 |
| PC1 score x Mismatch | 0.021 | [0.008, 0.034] | 0.001 | 0.006 | 9112.367 |
| Strength x Mismatch | -0.122 | [-0.141, -0.104] | 0.000 | 0.009 | 10189.081 |
| PC1 score x Strength x Mismatch | 0.014 | [-0.004, 0.033] | 0.123 | 0.009 | 10218.503 |

**Table F: PC5 scores as a function of Mismatch and Strength**

| **Fixed effects** | **Estimate** | **CI** | **p-value** | **SE** | **DF** |
| --- | --- | --- | --- | --- | --- |
| (Intercept) | 0.081 | [-0.353, 0.515] | 0.301 | 0.045 | 1.137 |
| Mismatch | -0.041 | [-0.094, 0.012] | 0.127 | 0.027 | 10258.839 |
| Strength | -0.090 | [-0.134, -0.046] | 0.000 | 0.023 | 10260.729 |
| Mismatch x Strength | 0.045 | [-0.033, 0.124] | 0.257 | 0.040 | 10255.146 |

**Table G: PC5 scores and RTs**

| **Fixed effects** | **Estimate** | **CI** | **p-value** | **SE** | **DF** |
| --- | --- | --- | --- | --- | --- |
| (Intercept) | -0.663 | [-1.107, -0.219] | 0.033 | 0.036 | 1.016 |
| PC5 score | 0.015 | [0.007, 0.022] | 0.000 | 0.004 | 221.355 |
| Strength | 0.215 | [0.205, 0.226] | 0.000 | 0.005 | 10252.177 |
| Mismatch | 0.163 | [0.151, 0.176] | 0.000 | 0.006 | 10232.294 |
| PC5 score x Strength | 0.010 | [-0.001, 0.020] | 0.066 | 0.005 | 9762.962 |
| PC5 score x Mismatch | -0.008 | [-0.021, 0.004] | 0.203 | 0.006 | 10076.592 |
| Strength x Mismatch | -0.127 | [-0.145, -0.108] | 0.000 | 0.009 | 10232.382 |
| PC5 score x Strength x Mismatch | -0.002 | [-0.021, 0.016] | 0.796 | 0.010 | 10158.572 |

**Table H: PC2 scores as a function of Mismatch and Strength**

| **Fixed effects** | **Estimate** | **CI** | **p-value** | **SE** | **DF** |
| --- | --- | --- | --- | --- | --- |
| (Intercept) | 0.030 | [-0.033, 0.092] | 0.349 | 0.031 | 114.669 |
| Mismatch | -0.051 | [-0.106, 0.004] | 0.069 | 0.028 | 10274.426 |
| Strength | -0.028 | [-0.074, 0.018] | 0.232 | 0.023 | 10278.012 |
| Mismatch x Strength | 0.066 | [-0.016, 0.147] | 0.113 | 0.041 | 10266.351 |

**Table I: PC2 scores and RTs**

| **Fixed effects** | **Estimate** | **CI** | **p-value** | **SE** | **DF** |
| --- | --- | --- | --- | --- | --- |
| (Intercept) | -0.658 | [-0.789, -0.526] | 0.005 | 0.020 | 1.430 |
| PC2 score | 0.007 | [-0.001, 0.014] | 0.077 | 0.004 | 258.877 |
| Strength | 0.208 | [0.193, 0.223] | 0.000 | 0.008 | 108.689 |
| Mismatch | 0.159 | [0.142, 0.176] | 0.000 | 0.009 | 140.013 |
| PC2 score x Strength | 0.008 | [-0.003, 0.018] | 0.163 | 0.005 | 9256.534 |
| PC2 score x Mismatch | 0.004 | [-0.008, 0.017] | 0.504 | 0.006 | 9766.249 |
| Strength x Mismatch | -0.127 | [-0.146, -0.109] | 0.000 | 0.009 | 10204.415 |
| PC2 score x Strength x Mismatch | -0.012 | [-0.031, 0.006] | 0.202 | 0.009 | 9982.026 |

**Table J: PC2 scores as a function of Mismatch, Strength, and Memory Accuracy**

| **Fixed effects** | **Estimate** | **CI** | **p-value** | **SE** | **DF** |
| --- | --- | --- | --- | --- | --- |
| (Intercept) | 0.022 | [-0.038, 0.083] | 0.464 | 0.031 | 126.781 |
| Mismatch | -0.050 | [-0.105, 0.005] | 0.073 | 0.028 | 12075.251 |
| Accuracy | -0.152 | [-0.291, -0.013] | 0.032 | 0.071 | 12122.422 |
| Strength | -0.024 | [-0.070, 0.022] | 0.303 | 0.023 | 12083.804 |
| Mismatch x Accuracy | 0.090 | [-0.119, 0.300] | 0.398 | 0.107 | 12147.439 |
| Mismatch x Strength | 0.061 | [-0.021, 0.142] | 0.144 | 0.042 | 12073.577 |
| Accuracy x Strength | 0.203 | [0.050, 0.355] | 0.009 | 0.078 | 12146.127 |
| Mismatch x Accuracy x Strength | -0.122 | [-0.371, 0.127] | 0.338 | 0.127 | 12115.314 |

**Table K: PC6 scores as a function of Mismatch and Strength**

| **Fixed effects** | **Estimate** | **CI** | **p-value** | **SE** | **DF** |
| --- | --- | --- | --- | --- | --- |
| (Intercept) | 0.020 | [-1.241, 1.281] | 0.878 | 0.103 | 1.015 |
| Mismatch | 0.085 | [0.033, 0.136] | 0.001 | 0.026 | 488.307 |
| Strength | -0.038 | [-0.094, 0.019] | 0.190 | 0.028 | 114.084 |
| Mismatch x Strength | 0.042 | [-0.030, 0.114] | 0.256 | 0.037 | 10240.374 |

**Table L: PC6 scores and RTs**

| **Fixed effects** | **Estimate** | **CI** | **p-value** | **SE** | **DF** |
| --- | --- | --- | --- | --- | --- |
| (Intercept) | -0.653 | [-0.879, -0.428] | 0.014 | 0.026 | 1.198 |
| PC6 score | -0.040 | [-0.052, -0.029] | 0.000 | 0.006 | 145.644 |
| Strength | 0.203 | [0.188, 0.218] | 0.000 | 0.008 | 105.857 |
| Mismatch | 0.158 | [0.141, 0.174] | 0.000 | 0.008 | 139.184 |
| PC6 score x Strength | 0.005 | [-0.006, 0.016] | 0.350 | 0.006 | 4707.178 |
| PC6 score x Mismatch | 0.015 | [0.002, 0.027] | 0.026 | 0.007 | 5396.250 |
| Strength x Mismatch | -0.124 | [-0.142, -0.105] | 0.000 | 0.009 | 10160.143 |
| PC6 score x Strength x Mismatch | -0.008 | [-0.026, 0.011] | 0.415 | 0.009 | 10218.930 |
