# Supplementary material for: Strong mnemonic prediction errors increase cognitive control, attention, and arousal
Source: eLife. 2026 Oct 21;15:RP109678. doi: 10.7554/eLife.109678 (PMC13686618; doi:10.7554/eLife.109678)
Supplement: Peer review history

# Editor report: eLife Assessment

**DOI:** 10.7554/eLife.109678.4.sa0

**Authors:** Lila Davachi

**Affiliations:** Yale University United States

**Keywords:** Important, Solid

The work presented represents **important** new evidence linking the cascade of neural processes triggered by memory-based prediction errors. The study uses an impressive collection of approaches and methods to characterize and measure cognitive control, arousal, and memory changes as a function of memory-based violations. The analyses are technically sophisticated and rigorous and, taken together, provide **solid** evidence that there are multiple processes accompanying prediction errors, and that they differentially relate to successful encoding.

# Referee report: Reviewer #1 (Public review)

**DOI:** 10.7554/eLife.109678.4.sa1

This manuscript describes a multi-modal study of associative learning and memory in humans that combines scalp EEG, pupillometry and behavioral analysis to explore the construct of mnemonic prediction errors (MPEs), in terms of their relationship to attention and cognitive control. Across two pooled studies, participants performed associative memory tasks in which they learned the relationship between a cue word (action verb) and subsequent picture (animate or inanimate) with a strong vs. weak (4 or 1 repetitions) encoding manipulation. At test, participants were encouraged to generate a prediction following the cue word to determine whether the subsequently presented picture was a match or mismatch. The timecourse of pupillary responses during match decisions were decomposed using temporal principal components analysis, which identified 6 distinct and overlapping processes. Some of the components (PC3/PC4) exhibited sensitivity to both the strength and mismatch conditions, as well as behavior (both RT and accuracy) and retrieval success on the subsequent trial. Furthermore, relationships were also observed between pupillary responses (specifically for PC4) and both frontal theta and posterior alpha power measures obtained from scalp EEG in Experiment 2, as well as for frontal theta and subsequent learning from mismatch stimuli (assessed using subsequent memory findings from a surprise recognition test). The authors suggest the findings indicate that MPEs elicit changes in attention, arousal and cognitive control which impact subsequent learning.

Strengths:

This manuscript has many strengths, including a clever study design, thoughtful integration of multiple neurocognitive measures, and a set of rigorous and technically sophisticated analyses, which reveal a large set of relationships among the measures and behavior. The findings demonstrating brain/physiology-behavior relationships are particularly important, in that they point to potential functional consequences of MPEs.\

# Referee report: Reviewer #2 (Public review)

**DOI:** 10.7554/eLife.109678.4.sa2

Summary:

The authors studied cognitive control and attention in response to mnemonic prediction errors (MPEs): situations in which the external reality violates internal memory-based predictions. The behavioral task first established strong versus weak predictions, and then either confirmed or violated these predictions. The authors examined markers of cognitive control (frontal theta) and attention (posterior alpha suppression, pupil response) while strong and weak predictions were confirmed or violated. They found increased cognitive control (frontal theta) for strong MPEs, which correlated with subsequent memory. Markers of attention (alpha suppression, pupil response) also accompanied strong MPEs but did not correlate with subsequent memory. Pupil response was investigated using an interesting approach that decomposes the response into different components, finding that different components respond earlier or later and show different correlations with MPEs and their strength. The authors also investigated how EEG, reaction time, and pupil responses correlated with one another, providing further insight into the mechanism underlying the response to MPEs. Together, the study points toward multiple control and attention mechanisms involved in MPE response and memory.

Strengths:

The study has a clear behavioral paradigm with multiple measures - behavioral, EEG, and pupillometry that offer an investigation into different aspects of MPE response and memory.

The study is also very comprehensive in looking at multiple phases in processing MPEs: the prediction phase (prior to the violation), the response to MPEs, and subsequent memory of MPEs, all within one study. Specifically, the link between neural mechanisms and subsequent memory is a major advancement, as most prior studies did not include this component. Mechanisms underlying subsequent memory of MPEs are theoretically important, as a primary function of MPEs is to promote learning and memory. As the authors mention, the different neural and pupillary signals are not robustly correlated, suggesting multiple mechanisms underlying MPE detections, which is interesting, offers avenues for future research, and can facilitate a better theory of how MPEs are processed in the brain. Finally, the decomposition of pupil response into different components and their correlation with behavior (RT during match/MPE detection) is interesting.

# Author comment: Author response

**DOI:** 10.7554/eLife.109678.4.sa3

**Authors:** Alice M Xue, Jenna Jokhani, Tony Norcia, Anthony D Wagner

**Affiliations:** Stanford University Stanford United States; Stanford University Stanford United States; Stanford University Stanford United States; Stanford University Stanford United States

The following is the authors’ response to the previous reviews.

**Note from the authors:**

During review of the proofs and re-confirmation of all models and outcomes, we discovered that in the current version of the manuscript, the identity of the lagged time series in the exploratory cross-correlation analysis was misreported. In the cross-correlation plot (Fig. 4d), the posterior alpha time series was fixed and the frontal theta time series was lagged by the times indicated on the x-axis, not vice versa. The inset figures depicting the timeseries will be corrected accordingly in the Version of Record. The subsequent mediation model, in which we tested whether attention mediates the relationship between mnemonic prediction strength and cognitive control, was included as a follow-up to the previously reported cross-correlation outcomes and will therefore be removed. As noted in the Version of Record, the corrected interpretation of the temporal ordering is that in response to a strong mnemonic prediction error, increases in cognitive control precede increases in attention.

**Public Reviews:**

**Reviewer #1 (Public review):**

Summary:

This manuscript describes a multi-modal study of associative learning and memory in humans, that combines scalp EEG, pupillometry and behavioral analysis to explore the construct of mnemonic prediction errors (MPEs), in terms of their relationship to attention and cognitive control. Across two pooled studies, participants performed associative memory tasks in which they learned the relationship between a cue word (action verb) and subsequent picture (animate or inanimate) with a strong vs. weak (4 or 1 repetitions) encoding manipulation. At test, participants were encouraged to generate a prediction following the cue word to determine whether the subsequently presented picture was a match or mismatch.

The timecourse of pupillary responses during match decisions were decomposed using temporal principal components analysis, which identified 6 distinct and overlapping processes. Some of the components (PC3/PC4) exhibited sensitivity to both the strength and mismatch conditions, as well as behavior (both RT and accuracy) and retrieval success on the subsequent trial. Furthermore, relationships were also observed between pupillary responses (specifically for PC4) and both frontal theta and posterior alpha power measures obtained from scalp EEG in Experiment 2, as well as for frontal theta and subsequent learning from mismatch stimuli (assessed using subsequent memory findings from a surprise recognition test). The authors suggest the findings indicate that MPEs elicit changes in attention, arousal and cognitive control which impact subsequent learning.

Strengths:

This manuscript has many strengths, including a clever study design, thoughtful integration of multiple neurocognitive measures, and a set of rigorous and technically sophisticated analyses, which reveal a large set of relationships among the measures and behavior. The findings demonstrating brain/physiology-behavior relationships are particularly important, in that they point to potential functional consequences of MPEs.

Weaknesses:

The technical proficiency and complexity of the study and analysis also presents a clear limitation and challenge for interpretation. It is likely that readers, even those that are quite knowledgeable about the methods, constructs, and questions being addressed will often struggle (as this reviewer did) to keep the large set of findings in mind and gain understanding of how they all fit together.

Indeed, it seems like there many threads running together in the paper which make it challenging to find the through-line of the key findings. The authors do address some of the key questions motivating the paper in the Introduction, but the results are somewhat ambiguous with regard to the primary question of the study as to whether the detection of MPEs leads to interaction among cognitive control, attention, and arousal. To their credit, the authors tackle this question through both cross-correlation and formal mediation analyses, and summarize these in diagrammatic figures (Figure 3, Figure 6). Yet it is not resolved whether the results represent a clear answer pointing to independence, or rather a lack of statistical power, or ill-resolved formulation of the mediational relationship. In particular, the cross-correlation suggests that posterior alpha suppression in response to MPEs does precede frontal theta, yet this indirect relationship does not explain the variation in trial-by-trial RTs on mismatches. This suggests a potential model misspecification.

In addition to the primary interaction issue mentioned above (between cognitive control, attention & arousal), the Introduction lays out a number of claims:

(1) That pupil size will be more sensitive to strong than weak MPEs.

(2) That MPE-linked increases in attention (indexed with posterior alpha suppression) and arousal (indexed with pupil size) will be linked to learning.

(3) That MPE learning will vary as a function of prediction strength.

Given the focus on learning, it is somewhat surprising that learning is not included in the mediation models. As the authors indicate in the Discussion, the use of trial-by-trial RT variation to drive the mediation model might be problematic, given that the RTs are sensitive to a range of factors beyond mnemonic prediction strength and also are under competing pressures (longer for mismatches than matches, due to surprise-linked slowing, but also faster following stronger rather than weaker mnemonic predictions). Thus, an alternative possibility might be to use trial-by-trial recognition of mismatches as the outcome variable in mediation models rather than trial-by-trial RT as the independent variable.

A large component of the results (Sections 2 and 3) is devoted to analyses of cue-linked pupil and EEG processes that putatively reflect mnemonic predictions (i.e., occurring before picture probes are presented and match/mismatch detection, i.e., MPEs occur). Yet these Results and the subsequent pupillary PCA components (PC1 and PC5) that are elicited are not well-integrated with the primary themes of the paper or the causal hypotheses. One finding that does seem to figure prominently (in that it is mentioned in Abstract, Introduction & Discussion) relates to the amount of attention allocated to the mnemonic prediction generation. Yet this finding is not well emphasized in the Results themselves. Possibly it refers to the negative relationship between posterior alpha during memory retrieval and the magnitude of pupillary PC3 component, described in Section 3. But it was quite challenging to identify amongst the wealth of results described in this Section as well as the others. More generally, the large amount of findings described across all four lengthy Results sections makes it challenging for readers to discern what are the key ones that the authors would like to highlight.

It is recommended that the authors do another pass through the paper to better highlight the most critical findings that they want to emphasize or which are most interpretable from a mechanistic and causal flow perspective and then de-emphasize or move other findings to the Supplemental Materials. Although the authors are to be commended for such a rigorous and comprehensive set of analyses, there are so many of them and findings, that the key points get buried and the reader needs to struggle potentially unnecessarily to identify the key take-away points.

We thank Reviewer 1 for the helpful feedback on how the manuscript can be further strengthened. We recognize that the rich set of findings can overwhelm the reader, resulting in difficulty discerning the main take aways about the effects of mnemonic prediction errors. We particularly appreciate Reviewer 1’s encouragement to restructure the manuscript so as to focus on the findings reported in Sections 1 and 4 of the original revision; the current revision now focuses on these key observations.

As part of this restructuring, Reviewer 1 also proposed moving the content from Sections 2 and 3 of the original revision to the Supplement. We agree with the Reviewer that the questions addressed in these sections on retrieval-related processes are not the main focus of the paper, but that they are informative in their own right. To avoid their getting lost in the Supplement, we decided that these results would be better served in a separate manuscript and thus we have removed them entirely.

We acknowledge in the revised manuscript that the mediation and cross-correlation analyses were exploratory and that these specific analyses may not be well powered in the current experiments. With respect to Reviewer 1’s concerns about the specification of the mediation models, we were motivated to test whether MPEs trigger an increase in cognitive control that in turn, triggers an increase in attention and/or arousal (Fig. 4a); as such, we designed the model to assess whether, on strong MPE trials, frontal theta mediates the relationship between prediction strength and attention/arousal. As noted in the manuscript and raised by Reviewer 1, mismatch RT here is an imperfect measure of trial-level prediction strength. Future experiments that selectively elicit strong MPEs and have a more controlled measure of trial-level prediction strength may be better equipped to address these questions about interactions between control, attention, and arousal. We agree with Reviewer 1 that models assessing subsequent memory as an outcome would be desirable. However, given that (a) we did not find strong evidence for interactions at the time of a strong MPE and (b) we only observed a relationship between frontal theta and subsequent memory (but not posterior alpha or pupil), subsequent memory mediation models do not appear to be well justified. Altogether, these findings illuminate open avenues for future research.

**Reviewer #2 (Public Review):**

Summary:

The authors studied cognitive control and attention in response to mnemonic prediction errors (MPEs): situations in which the external reality violates internal memory-based predictions. The behavioral task first established strong versus weak predictions, and then either confirmed or violated these predictions. The authors examined markers of cognitive control (frontal theta) and attention (posterior alpha suppression, pupil response) while strong and weak predictions were confirmed or violated. They found increased cognitive control (frontal theta) for strong MPEs, which correlated with subsequent memory. Markers of attention (alpha suppression, pupil response) also accompanied strong MPEs but did not correlate with subsequent memory.

Pupil response was investigated using an interesting approach that decomposes the response into different components, finding that different components respond earlier or later and show different correlations with MPEs and their strength. The authors also investigated how EEG, reaction time, and pupil responses correlated with one another, providing further insight into the mechanism underlying the response to MPEs. Together, the study points toward multiple control and attention mechanisms involved in MPE response and memory.

Strengths:

The study has a clear behavioral paradigm with multiple measures — behavioral, EEG, and pupillometry — that offer an investigation into different aspects of MPE response and memory.

The study is also very comprehensive in looking at multiple phases in processing MPEs: the prediction phase (prior to the violation), the response to MPEs, and subsequent memory of MPEs, all within one study. Specifically, the link between neural mechanisms and subsequent memory is a major advancement, as most prior studies did not include this component. Mechanisms underlying subsequent memory of MPEs are theoretically important, as a primary function of MPEs is to promote learning and memory. As the authors mention, the different neural and pupillary signals are not robustly correlated, suggesting multiple mechanisms underlying MPE detections, which is interesting, offers avenues for future research, and can facilitate a better theory of how MPEs are processed in the brain. Finally, the decomposition of pupil response into different components and their correlation with behavior (RT during match/MPE detection) is interesting.

Weaknesses:

The methods are rigorous, and the data support the claims. The weaknesses are minor and are offered here as avenues for future research.

(4) The relationships the authors find between brain measures and pupil components were largely not specific to mismatches/matches. Thus, the specificity of this relationship is untested.

(5) The results with subsequent memory are important and address a major gap in the field that largely did not relate neural effects of MPE to subsequent memory. However, one major limitation of the study is that the authors did not test memory for matches. I understand the logic of avoiding testing matches. Because matches were repeated more times in the study, it’s not a fair comparison and could change participants’ overall criterion for old/new decisions. Future research could address this, e.g., by testing weak matches or potentially using a between-subject design.

We appreciate Reviewer 2’s helpful feedback during the review process and encouraging comments on the strengths of the manuscript. We agree and note in the revision that it would be illuminating for future studies to contrast memory for events that violate and confirm mnemonic predictions.

Comments on revised version

The authors addressed all my concerns. I appreciate the authors’ thoughtful and detailed response.

**Recommendations for the authors:**

**Reviewer #1 (Recommendations for the authors):**

It was challenging to read the paper with key findings happening at the time of the MPE presented first, and then to go “backwards in time” to examine process that occurred at preceding time periods (i.e., prior to probe presentation), and then again forward in time to examine learning related processes.

(1) Restructuring the Results

In this regard two distinct recommendations are made:

- Move Sections 2 and 3 to Supplemental Materials, to maintain the focus on the key findings related to detection of MPEs and their effects on subsequent learning.

- An alternative structure would be to present the cue-locked findings first, which relate to mnemonic predictions, then to those that occur when those predictions get violated, and finally to the learning process that occur following MPEs and which can be detected with subsequent recognition tests.

We thank the Reviewer for this encouragement to restructure the manuscript. To address concerns regarding the density of the paper and the cohesiveness of the findings, we removed the content that was in Sections 2 and 3 of the original revision. We will publish those results in a separate paper with additional analyses to more directly address previously raised questions regarding the mechanisms indexed by frontal theta and posterior alpha at the time of retrieval.

(2) Integration of the summary figures

At the minimum, a recommendation would be to better integrate Figure 6, and maybe various versions of Figure 3, earlier into the text, preferably even in the Introduction, and then repeatedly refer to them throughout the results. For example, in Figure 6, linking the leftmost panel of the figure to Sections 2 and 3 is critical, and the righthand panel to Sections 1 with the rightmost part related to learning explicitly linked to Section 4.

We moved the summary figure up to now be Figure 1; we reference this figure in the Introduction and throughout the manuscript; and we additionally make reference in the figure to the association between frontal theta and PC3 at the time of a strong MPE as well as the cross-correlation outcome. We hope these modifications further aid the reader in identifying the main findings of the manuscript.

(3) Specification of the mediation models

Additionally, for the mediation models it is quite unclear why mismatch RT is treated as the index of MPE magnitude, as this seems to be where the problem may lie in model fitting. Why not think of this as an outcome variable (since would seem to be a causal outcome of the underlying functional processes elicited by mismatch detection)?

We thank the reviewer for these thoughtful comments. Our goal in designing the mediation models in Fig. 4a-c was to test our hypothesis that strong MPEs trigger an increase in cognitive control that, in turn, triggers an increase in attention and/or arousal; thus, attention/arousal should be the outcome and cognitive control should be the mediator. Given that increases in attention and cognitive control were selectively observed for strong and not weak MPEs, the models were restricted to strong MPEs. We therefore needed a trial-level measure of MPE magnitude to determine whether stronger MPEs in the strong condition elicit greater attention/arousal by engaging more cognitive control. As such, we decided to use mismatch RT as a proxy measure of MPE magnitude; we acknowledge in the text that this measure is imperfect. While a model with mismatch RT as the outcome variable is possible, the relative timing of the attention/arousal effects (which largely occur following responses) would render interpretation to be more challenging. Had stronger evidence of indirect effects emerged in Figs. 4b-c, we could have conducted model comparison with RT as an outcome. We acknowledge in the text that these analyses were exploratory and characterization of potential indirect effects will require more data and a more precise measure of MPE magnitude.

Alternatively, examining trial-by-trial recognition memory of mismatches as the relevant outcome variable would also seem to capture the functional process of interest. In this regard, have the authors examined whether trial-by-trial RT on mismatches predicts subsequent recognition of these items? If this direct relationship holds, it could be a target for mediation analyses in itself.

We agree with the reviewer that in theory, a mediation model predicting subsequent memory would be a desirable test of an integrated model of the mechanisms underlying MPE-driven learning. However, the mediation analyses conducted to address the functional relationships at the time of a prediction error (Fig. 4) are not well-powered to begin with; this limitation is raised in the Results and the Discussion. A mediation model predicting subsequent memory would be similarly underpowered; given that there is not strong evidence for indirect effects at the time of a strong MPE, and that only frontal theta – and not posterior alpha nor pupil – predicts subsequent memory, we think that such a mediation model is not well justified to include. Such a model should be more directly tested by well-powered designs in future studies.

(4) Hippocampal theta in the Introduction

The Introduction discusses hippocampal theta as well as frontal theta yet also makes clear that the former is not really well-detected or analyzed using scalp EEG. Consequently, a recommendation would be to remove this paragraph from the Introduction, since it can be misleading and a “red herring” for the reader, and instead only bring up this point in the Discussion section, as a pointer to the need for future research using methods that may be more sensitive to hippocampal interactions with PFC regions.

We appreciate this point and moved discussion of the hippocampus from the Introduction to the Discussion.
